# Proximal-type epithelioid sarcoma: a new case report and literature review

**DOI:** 10.11604/pamj.2016.24.238.8535

**Published:** 2016-07-14

**Authors:** Abdelmoughit Echchaoui, Yassine Sadrati, Youssef Elbir, Abderrahim Elktaibi, Malika Benyachou, Samir El Mazouz, Nour-eddine Gharib, Abdellah Abbassi

**Affiliations:** 1 Department of Plastic Surgery and Burns, Avicenna University Hospital, Rabat, Morocco; 2 Department of Orthopaedic Surgery & Traumatology, Ibn Sina University Hospital, Rabat, Morocco; 3 Department of Pathology, Avicenna Military Hospital, Marrakech, Morocco

**Keywords:** Epithelioid sarcoma, proximal-type, diagnosis, prognosis

## Abstract

Proximal-type epithelioid sarcoma is a rare soft tissue neoplasm which arises from the more proximal part of body and occurs more often in young people; the definite diagnosis depends mainly on the pathological examination; early detection and complete excision remain the foundation of treatment. Due to its aggressive behavior, high capacity of recurrence and the great ability to metastasize, a careful clinical long-term monitoring is required. We report a new case of a 20 years old girl, presented with proximal-type epithelioid sarcoma in her right scapular region, confirmed by pathological examination and removed surgically without recurrence or metastasis at eighteen months of follow-up.

## Introduction

Proximal-type epithelioid sarcoma (PES) is a rare high grade soft-tissue sarcoma (less than 1 of all soft tissue sarcomas), typically presenting as a subcutaneous or deep dermal mass, with slow growing and high potential for distant metastasis in adolescents and young adults [1]. These tumors arise in proximal locations (trunk, axilla, genital area…), have a much worse prognosis if delayed diagnosis and treatment. Owing to its rarity, aggressive behavior, high capacity of recurrence and the difficulty of its clinical and histopathological diagnosis, we report a new case of a 20 years old girl, presented with proximal-type epithelioid sarcoma in her right scapular region removed and treated successfully.

## Patient and observation

**Figure 1:**
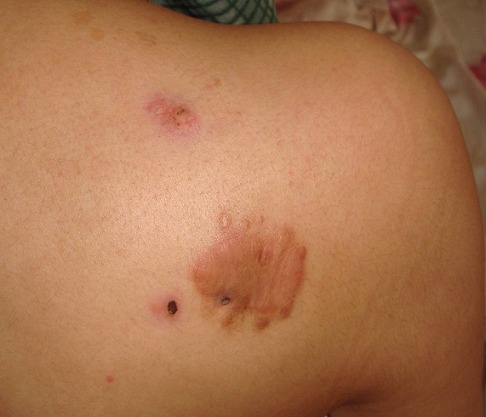
The two farms painless nodules and a solid plaque on the right scapular region

**Figure 2:**
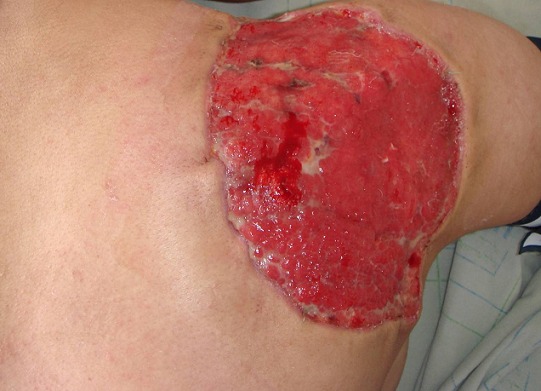
A loss of substance in granulation after wide excision

**Figure 3:**
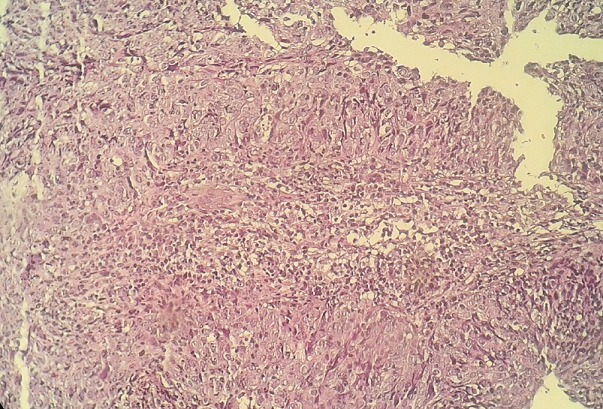
Microscopic appearance of the surgical specimen showing weakly eosinophilic epithelioid and elongated cells exhibiting slight nuclear atypia (Hematoxylin and Eosin × 250)

**Figure 4:**
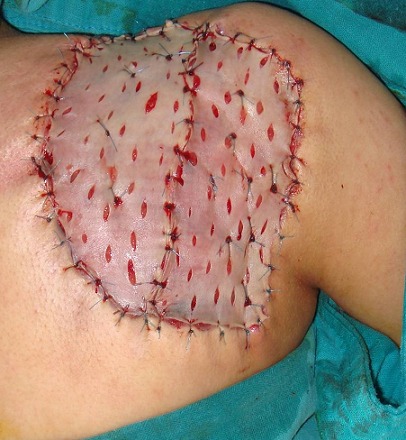
Coverage of the loss of substance by graft of thin skin

A 20 years old girl presented with a red and farm nodule since 2 years in her right scapular region; a first simple excision was performed and was in favor of a benign lesion (fibrous histiocytoma). The lesion recurred clinically six months later as two farms painless nodules and a solid plaque measuring 3.5cm in diameter (Figure 1). There was no lymphadenopathy. The rereading of the histopathological slides made the diagnosis of superficial subcutaneous epithelioid sarcoma and a chest and abdominal computed tomography ruled out metastasis. Wide excision with surgical margins of 4 cm was performed taking the deep fascial plane (Figure 2). The histological examination confirmed the diagnosis with complete resection limits (Figure 3). In immunohistochemical staining, the tumor cells were immunoreactive for vimentin and epithelial markers (cytokeratin, CD34). The loss of substance left in granulation for a few days, was grafted by thin skin (Figure 4). There was no recurrence or metastasis at eighteen months of follow-up.

## Discussion

Epithelioid sarcoma was first described by Enzinger in 1970 [2]; usually arises in the distal extremities in adolescents and young adults with male prevalence; the proximal-type variant, first described in 1997 as an aggressive form of sarcoma with a great ability to metastasize and high capacity of recurrence, usually arises more proximally [3]. Proximal-type epithelioid sarcomas (PES), occur 94% in the chest wall, inguinal region, thigh and perineum, Its size at presentation varies from 0.5 to 19 cm [4], and growth duration ranges from 6 months to 5 years [5]. This uncommon neoplasm is a pitfall in clinical diagnosis and it is likely to be confused with benign lesions resulting in delayed diagnosis and treatment [6]. PES can be diagnosed only through histological examination [7] showing a pleomorphic epithelioid and oval spindled malignant cells with numerous mitoses exhibiting slight nuclear atypia, vesicular nuclei and small nucleoli, transition between the two cell types is gradual and intercellular collagen deposition usually marked [4, 8]; it can be distinguished from poorly differentiated carcinoma, rhabdomyosarcoma, synovial sarcoma, malignant mesothelioma, and melanoma using immunohistochemistry [9]. Immunohistochemically, PES is characteristically immunoreactive for vimentin and epithelial markers: low and high molecular weight cytokeratins, keratin 8, keratin 19 and/or EMA. Half of the cases are also positive for CD34 [10]. Several publications about misdiagnosis and prognosis of PES have been raised currently. Fisher et al [11] emphasize the importance of using immunohistochemicals markers to the diagnostic of specific soft tissue tumors specially co-expression of CD34 and cytokeratins in PES cells. Mannan et al [12] report a case of PES in a 47-year-old man, to emphasize the importance of diagnosing of this challenging tumor and the role of immunohistochemistry in establishing the diagnosis. Wide local excision with adequate margins (at least 2 cm) is recommended [13]; adjuvant radiotherapy is advocated in high-grade tumors or inadequate surgical margins [14], and also due to high incidence of local recurrence and distant metastasis [10], However it is controversial and it did not show statistically significant reduction in mortality [15]. The role of chemotherapy in the adjuvant setting appears marginally effective at best in the treatment of metastatic disease [16]. The prognosis for PES remains worse; despite negative surgical margins, tendency to recur is highly possible and distant metastasis eventually occurs in up to 60% of cases [14] Ulbright et al. [17] reported in their review of the literature that all patients with local recurrence ultimately died from distant metastasis. Our patient had initially a 2- years history of a progressively growing nodule over the right scapular region, which could be considered as a proximal type variant. As the lesion was recurrent a wider excision with surgical margins of 4 cm was performed. The histological examination and immunohistochemistry confirmed the diagnosis of PES. She underwent no postoperative adjuvant therapy and she is well without recurrence at eighteen months of follow-up.

## Conclusion

PES is a rare disease with aggressive behavior and poor prognosis. Early detection, radical surgical excision and histopathological examination are crucial to provide a chance of a cure. The wide variability of clinical expressions and totally unpredictable evolutionary nature require regular long-term monitoring in detecting local recurrence and distant metastasis.
